# Sleep Quality as a Predictor of Coronary Artery Disease Severity in Geriatric Acute Coronary Syndrome

**DOI:** 10.3390/medicina62010101

**Published:** 2026-01-02

**Authors:** Hasan Can Konte, Emir Dervis, Omer Alyan, Dursun Aras

**Affiliations:** Department of Cardiology, Istanbul Medipol University, Istanbul 34815, Turkey; emirdervis@hotmail.com (E.D.); droalyan@yahoo.com (O.A.); drdaras@gmail.com (D.A.)

**Keywords:** acute coronary syndrome, sleep wake disorders, aged, coronary artery disease, cardiac catheterization

## Abstract

*Background and Objectives:* The conflicting findings in existing studies and insufficient evidence highlight the necessity for additional research on the relationship between sleep quality and coronary artery disease (CAD) in elderly acute coronary syndrome (ACS) patients. We aimed to investigate the association between sleep quality and the CAD severity of in geriatric patients with ACS. *Materials and Methods:* This retrospective observational cohort study analyzed data from 308 patients aged 65 years and older admitted with ACS who had undergone coronary angiography between May 2022 and June 2025 at a tertiary cardiology department. Sleep quality was assessed using the Pittsburgh Sleep Quality Index (PSQI) at the 6-month follow-up, with scores > 5 indicating poor quality. CAD severity was quantified by SYNTAX score from angiograms. The primary endpoint was the relationship between PSQI and SYNTAX score, with secondary analyses concerning factors associated with clinical outcomes. *Results:* Poor sleep quality (PSQI > 5) was associated with higher SYNTAX scores (*p* < 0.001), lower ejection fraction (*p* < 0.001), higher CRP (median 5.1 vs. 4.05, *p* = 0.029), NT-proBNP (median 748.5 vs. 595, *p* = 0.034), lower glomerular filtration rate (*p* = 0.025), and higher hypertension prevalence (*p* = 0.034). ST-elevation myocardial infarction was more common in subjects with poor sleep. Multivariable logistic regression identified hypertension (*p* = 0.011), reduced ejection fraction (*p* = 0.030), STEMI (*p* = 0.045), intermediate SYNTAX (*p* = 0.003), and high SYNTAX (*p* = 0.009) as associated factors of poor sleep. *Conclusions:* Poor sleep quality is independently linked to greater CAD severity in geriatric ACS patients. This is a modifiable risk factor that can reduce morbidity and mortality in this high-risk group.

## 1. Introduction

Acute coronary syndrome (ACS) is the development of acute myocardial ischemia causing reduced coronary blood flow. Conditions described within the definition of ACS are ST-elevation myocardial infarction (STEMI), non-ST-elevation myocardial infarction (NSTEMI), and unstable angina pectoris (USAP) [1]. ACS accounts for approximately 7 million hospitalizations every year and is the leading cause of cardiovascular mortality [2]. Its incidence rises sharply with age; in individuals aged 65 years and older, ACS prevalence is estimated at 20–30 per 1000 person-years, compared to less than 5 per 1000 in younger adults [3]. Geriatric patients face heightened risks due to comorbidities such as hypertension, diabetes, and reduced physiological reserve [4]. The mechanisms contributing to increased severity include endothelial dysfunction, chronic inflammation, and polypharmacy, which require careful consideration of risks in this population [5].

Sleep quality, defined as the subjective and objective adequacy of sleep continuity, depth, and restorative effects, is integral to overall health, while sleep disorders such as insomnia, obstructive sleep apnea (OSA), and fragmented sleep disrupt circadian rhythms and homeostasis [6]. Poor sleep quality affects 30–50% of the general population and is linked to adverse health outcomes, including hypertension, metabolic syndrome, and cognitive decline [7]. The American Heart Association has recognized sleep health as an essential component of cardiovascular health, with sleep duration and quality playing critical roles in cardiometabolic disease prevention [8]. This scientific statement highlights the importance of assessing sleep as a modifiable cardiovascular risk factor, particularly in high-risk populations such as elderly patients with established coronary artery disease. In the elderly, sleep disturbances are particularly prevalent, impacting up to 50–70% of individuals over 65 years due to age-related changes, such as reduced slow-wave sleep, increased awakenings, and circadian misalignment [9]. These changes may contribute to greater levels of cardiovascular risk due to sympathetic activation, endothelial injury due to oxidative stress, and overall inflammatory activity, which can be associated with sleep quality [10,11].

Emerging evidence suggests a bidirectional association between sleep quality, sleep disorders, and ACS. Large prospective cohort studies, including the Multi-Ethnic Study of Atherosclerosis (MESA) and the Nurses’ Health Study, have demonstrated significant associations between sleep duration, sleep quality, and incident cardiovascular events [12,13]. Poor sleep quality and short sleep duration (<6 h) have been associated with increased coronary artery calcification, arterial stiffness, and coronary artery disease (CAD) progression, potentially exacerbating ACS severity via endothelial dysfunction and plaque instability [14,15]. These cohort studies provide compelling evidence that sleep disturbances precede cardiovascular events, supporting the potential role of sleep quality as a modifiable risk factor in cardiovascular disease prevention.

OSA, characterized by recurrent apneic events, correlates with higher SYNTAX scores and adverse ACS outcomes, including elevated troponin and NT-proBNP levels [16,17]. However, studies in geriatric populations have described contrasting findings. Some report protective effects of mild sleep disturbances via ischemic preconditioning [18], while others show increased risks including major adverse cardiovascular events (MACE) among elderly ACS patients with poor sleep [12].

The inconsistencies in available literature and the limited evidence indicate the need for further investigations examining how sleep and its quality can impact CAD among older patients with ACS. We aimed to evaluate the association between sleep quality, assessed by the Pittsburgh Sleep Quality Index (PSQI) at 6 months post-ACS, and CAD severity measured by SYNTAX score in geriatric ACS patients.

## 2. Materials and Methods

### 2.1. Ethical Issues

This retrospective observational study received ethical approval from the Ethics Committee of Istanbul Medipol University (Approval date: 25 September 2025, no: 1183). Given the retrospective design, informed consent was waived in accordance with institutional policies. All procedures complied with the ethical standards of the Declaration of Helsinki and relevant national regulations.

### 2.2. Study Design and Population

This was designed as a retrospective, observational cohort study. Data were collected from patients presenting with ACS who underwent coronary angiography between May 2022 and June 2025 at the Department of Cardiology of Istanbul Medipol University Hospital, Istanbul, Turkey.

The study population comprised geriatric patients aged 65 years and older who were admitted with a diagnosis of ACS and underwent coronary angiography during the specified period. Inclusion criteria were as follows: adult patients aged 65 years or older with confirmed ACS; at least 6 months of follow-up post-procedure; PSQI assessment recorded at the 6-month follow-up visit; and cognitive adequacy (Mini-Mental State Examination score ≥ 24 or Montreal Cognitive Assessment score ≥ 26) to ensure reliable self-reporting. Exclusion criteria included known sleep disorders under treatment (e.g., insomnia, restless legs syndrome, narcolepsy); active psychiatric conditions (e.g., major depression, anxiety disorder); shift workers or those with irregular sleep schedules; use of sedative/hypnotic medications; dementia or severe cognitive impairment; diagnosed obstructive sleep apnea (OSA) or those receiving continuous positive airway pressure therapy; alcohol or substance dependence; severe valvular pathologies; malignant arrhythmias; malignancy; New York Heart Association class IV heart failure; end-stage renal disease; follow-up duration less than 1 year; or recurrent acute myocardial infarction within the last 6 months. Patients who refused coronary angiography were also excluded.

Sample size determination was based on an a priori power analysis using G*Power software (version 3.1.9.7; Heinrich Heine University, Düsseldorf, Düsseldorf, Germany), drawing from values reported by Lao et al. [4], where the effect size was estimated at 0.070. With a Type I error (α) of 0.05 and power (1−β) of 0.80, a minimum sample size of 308 participants was calculated as appropriate to detect significant associations. This sample size provides adequate statistical power for the planned analyses.

### 2.3. Medical Records and Data Handling

Data collection involved a comprehensive review of demographic, clinical, laboratory, and procedural variables from electronic health records, patient charts, and follow-up clinic notes. Variables were categorized into sociodemographic and clinical characteristics, laboratory measurements, sleep characteristics, and ACS management details.

### 2.4. Sociodemographic and Clinical Data

Sociodemographic data included age, sex, body mass index (BMI, calculated as weight in kg divided by height in m^2^), living status (e.g., living alone), and smoking history (current smoker or nonsmoker). Examined comorbidities included hypertension, diabetes mellitus, stroke, atrial fibrillation, thyroid disease, and chronic obstructive pulmonary disease.

Cardiovascular parameters included left ventricular ejection fraction (LVEF, assessed by echocardiography using Simpson’s method), medication use (e.g., diuretics, angiotensin-converting enzyme inhibitors, angiotensin receptor blockers, beta-blockers, dihydropyridine calcium channel blockers), and ACS subtype [NSTEMI, STEMI, or USAP, classified according to electrocardiographic and biomarker criteria as per European Society of Cardiology guidelines [1]. Revascularization strategies were recorded as medical management, percutaneous coronary intervention, or coronary artery bypass grafting.

### 2.5. Sleep Quality Assessment

Sleep quality was evaluated using the Turkish version of the PSQI [19], a validated self-report questionnaire administered at the 6-month follow-up visit. This timing ensured that sleep quality assessment reflected stable conditions after patients had completed their post-ACS recovery phase rather than acute illness effects, and all assessments were conducted after coronary angiography and revascularization procedures had been completed. The PSQI assesses sleep over the preceding month across seven components: subjective sleep quality, sleep latency, sleep duration, habitual sleep efficiency, sleep disturbances, use of sleeping medications, and daytime dysfunction. Each component is scored from 0 to 3, yielding a global PSQI score ranging from 0 to 21, with higher scores indicating poorer sleep quality. A global score > 5 was considered indicative of poor sleep quality, as established in prior studies [6,9]. Sleep latency was categorized as <15 min, 16–30 min, 31–60 min, or >60 min; sleep duration as >7 h, 6–7 h, 5–6 h, or <5 h; and sleep efficiency as ≥85%, 75–84%, 65–74%, or <65% (calculated as [hours slept/hours in bed] × 100). The PSQI has been extensively validated in cardiovascular populations and demonstrates high sensitivity (89.6%) and specificity (86.5%) for distinguishing good from poor sleepers. The questionnaire assesses sleep patterns over the preceding month, providing a comprehensive evaluation of chronic sleep quality rather than transient sleep disturbances.

Patients with diagnosed obstructive sleep apnea or those receiving continuous positive airway pressure therapy were excluded from this study. However, systematic screening for undiagnosed OSA using polysomnography or home sleep apnea testing was not performed. While clinical records were reviewed for documented OSA risk factors such as witnessed apneas, loud snoring, and excessive daytime sleepiness, the absence of systematic OSA screening represents a limitation, as undetected sleep-disordered breathing could potentially confound the observed associations between sleep quality and CAD severity.

### 2.6. Coronary Artery Disease Management and Severity Assessment

The management of ACS was conducted in accordance with the current ACC/AHA/ACEP/NAEMSP/SCAI Guideline for the Management of Patients With ACS. The extent and severity of CAD were quantified using the SYNTAX score, calculated from coronary angiography images by two blinded interventional cardiologists using the online SYNTAX calculator (version 2.11) [20]. The SYNTAX score accounts for lesion complexity, location, and functional impact, with scores categorized as low (≤22), intermediate (23–32), or high (≥33). Inter-observer reliability was assessed using Cohen’s kappa coefficient, yielding substantial agreement (κ = 0.78). In cases of score discrepancy >5 points, a third senior interventional cardiologist adjudicated, and consensus was reached through discussion.

### 2.7. Laboratory Measurements

Laboratory data were obtained from fasting venous blood samples collected at admission. Parameters included hemoglobin, platelet count, white blood cell count, creatinine with estimated glomerular filtration rate [eGFR, using the CKD-EPI equation [21], uric acid, C-reactive protein (CRP), total cholesterol, high-density lipoprotein, low-density lipoprotein, triglycerides, fasting blood glucose, peak troponin, and N-terminal pro-brain natriuretic peptide (NT-proBNP). These biomarkers were analyzed using automated analyzers (Roche Diagnostics, Basel, Switzerland) in our Biochemistry Department.

### 2.8. Grouping and Endpoints

Patients were grouped based on sleep quality: good sleep quality (PSQI global score ≤ 5) and poor sleep quality (PSQI global score > 5), as per validated cutoffs [6]. The primary endpoint was the association between sleep quality (measured by PSQI at 6 months) and CAD severity (assessed by SYNTAX score), with secondary explorations of relationships with clinical outcomes.

### 2.9. Statistical Analysis

All analyses were conducted using IBM SPSS version 27.0 (IBM Corp., Armonk, NY, USA). *p* < 0.05 values were accepted as statistically significant results. Normality assumption was evaluated using the histograms and Q-Q plots. Descriptive statistics were presented using mean ± standard deviation for normally distributed continuous variables, median (25th percentile–75th percentile) for non-normally distributed continuous variables and frequency (percentage) for categorical variables. Between groups analysis of continuous variables were performed using Student’s t test or Mann–Whitney U test depending normality of distribution. Between groups analysis of categorical variables were performed using the chi-square test or Fisher’s exact test. Multivariable logistic regression analysis was performed to determine significant factors independently associated with high (>5) PSQI score. Variable selection for the multivariable model was based on both a priori clinical relevance and univariate associations with the outcome. Variables with *p* < 0.10 in univariate analysis were considered for inclusion. The final model included age, hypertension, left ventricular ejection fraction, ACS type (STEMI, NSTEMI, USAP), SYNTAX score category (low, intermediate, high), estimated glomerular filtration rate, C-reactive protein, and NT-proBNP. The rationale for including these covariates was based on their established associations with both sleep disturbances and cardiovascular outcomes in prior literature, as well as their potential to confound the relationship between sleep quality and CAD severity. Collinearity among independent variables was assessed using variance inflation factors (VIF), with VIF > 5 indicating problematic multicollinearity. All VIF values were <3, indicating acceptable collinearity. Results are presented as odds ratios (OR) with 95% confidence intervals (CI)

## 3. Results

**Figure 1:**
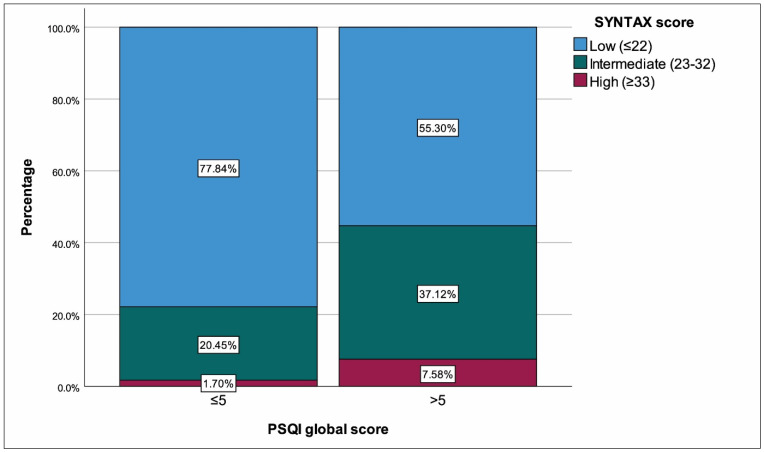
SYNTAX scores with regard to PSQI score.

The study included a total of 308 geriatric patients with ACS. The median PSQI global score was 5 (interquartile range 3–7, range 0–19). Patients were divided into two groups based on PSQI scores: good sleep quality (≤5, n = 176, 57.14%) and poor sleep quality (>5, n = 132, 42.86%). As anticipated, all parameters linked to poor quality sleep were more common among patients with a PSQI score of >5. Overall, the cohort was predominantly male (n = 283, 91.88%). There was no significant difference in sex distribution between the groups (*p* = 0.609). The median age was 67 years (65–73) overall, and patients with poor sleep quality were marginally older (*p* = 0.049). Hypertension was more prevalent in the poor sleep quality group than in the good sleep quality group (*p* = 0.034). Mean LVEF was lower in the poor sleep quality group compared to the good sleep quality group (*p* < 0.001). ACS subtypes differed significantly (*p* = 0.016), with STEMI more common in the poor sleep quality group and USAP more common in the good sleep quality group. SYNTAX scores were higher in the poor sleep quality group (*p* < 0.001), with a greater percentage of intermediate and high scores in the poor sleep quality group compared to low scores (*p* < 0.001) (Figure 1). Laboratory data showed lower eGFR (*p* = 0.025), higher CRP (*p* = 0.029), and higher NT-proBNP (*p* = 0.034) in patients with poor sleep (Table 1).

Multivariable logistic regression showed that hypertension (OR: 1.971, 95% CI: 1.168–3.328, *p* = 0.011), low LVEF (OR: 0.949, 95% CI: 0.905–0.995, *p* = 0.030), STEMI (OR: 2.151, 95% CI: 1.019–4.542, *p* = 0.045), intermediate (23–32) SYNTAX score (OR: 2.347, 95% CI: 1.350–4.082, *p* = 0.003) and high (≥33) SYNTAX score (OR: 6.336, 95% CI: 1.573–25.514, *p* = 0.009) were independently associated with high (>5) PSQI score (Table 2). The highest VIF was 2.636 (for ejection fraction), means no multicollinearity in the regression model.

Patients were further stratified by SYNTAX score into low (n = 210, 68.18%) and intermediate/high (n = 98, 31.82%) groups. A PSQI of ≤5 was more prevalent in the low SYNTAX group than in the intermediate/high group (*p* < 0.001). Sleep duration differed significantly (*p* = 0.004), with >7 h more common in the low SYNTAX group and 5–6 h more prevalent in the intermediate/high group. Sleep efficiency was significantly lower in the intermediate/high SYNTAX group (*p* < 0.001) (Table 3).

## 4. Discussion

The current study revealed that in geriatric patients with ACS, poor sleep quality, as assessed at six months post-event, was significantly associated with greater CAD severity. Additionally, patients exhibiting poor sleep quality demonstrated a higher prevalence of hypertension, reduced LVEF, elevated inflammatory and cardiac stress biomarkers such as CRP and NT-proBNP, and a greater likelihood of presenting with STEMI compared to unstable angina. Patients with intermediate/high SYNTAX scores had significantly poorer sleep quality, shorter sleep duration, and lower sleep efficiency than those with low SYNTAX scores. An important methodological consideration is the cross-sectional nature of the association observed in this study. While our findings demonstrate that poor sleep quality correlates with greater CAD severity, the directionality of this relationship cannot be determined from our data. It is equally plausible that more severe coronary atherosclerosis leads to poor sleep quality through mechanisms such as nocturnal angina, orthopnea, autonomic dysfunction, or increased use of medications that disrupt sleep architecture. Indeed, the relationship between sleep disturbances and cardiovascular disease is likely bidirectional, with each condition potentially exacerbating the other. Therefore, our results should be interpreted as demonstrating an association rather than establishing sleep quality as a predictor or causal factor in CAD severity.

Chronic sleep disturbances disrupt autonomic balance and promote inflammatory pathways, thereby accelerating CAD progression and heightening vulnerability to ACS in susceptible individuals [22,23]. In elderly populations, where physiological changes such as diminished melatonin secretion and fragmented sleep phases are common, these disruptions can exacerbate existing vascular vulnerabilities [7,9]. In our study, geriatric ACS patients with poor sleep quality (PSQI > 5) exhibited significantly higher SYNTAX scores, indicating more complex and extensive coronary lesions, compared to those with good sleep quality. This finding aligns with prior research. Yakut et al. reported that baseline coronary artery stenosis severity, quantified by SYNTAX score, independently predicted subsequent poor sleep quality in ACS patients, with a threefold increased risk of poor sleep quality among patients with SYNTAX of >22 [2]. Similarly, Yang et al. observed that poor sleep quality correlated with elevated SYNTAX scores and worse prognosis in young ACS patients, suggesting a consistent pattern across age groups, though potentially more pronounced in the elderly due to comorbidities [5]. In a prospective cohort by Zeng et al., OSA combined with incomplete revascularization (residual SYNTAX score > 0) heightened major adverse cardiovascular and cerebrovascular events in ACS patients [3]. However, Jia et al. found a link between OSA and CAD progression but not sleep quality metrics [17]. We demonstrate this association specifically in geriatric ACS survivors using PSQI at six months. Although bidirectional relationships should be considered in this respect, it is crucial to recognize that poor sleep quality is largely a modifiable factor that may indeed achieve improvements in cardiac prognosis among elderly patients with ACS. Poor sleep quality can contribute to CAD severity through elevated cortisol levels, alterations in nitric oxide levels, oxidative stress, and the concerted impacts on plaque vulnerability [1,4,24].

Compensatory mechanisms in heart functioning are often impaired by age-related declines in baroreflex sensitivity and cardiac reserve [25,26]. Sleep disorders trigger nocturnal hypoxia and arousal-induced surges in blood pressure, which can cause plaque rupture [14,15]. In our results, poor sleep quality was associated with a higher incidence of STEMI and elevated levels of biomarkers like NT-proBNP and CRP, suggesting greater myocardial stress and inflammation. This is consistent with findings from Calcaianu et al., who in a prospective study of ACS patients with OSA, reported that an apneic coefficient (apnea index/apnea-hypopnea index) ≥ 37% correlated with higher troponin, NT-proBNP, and lower ejection fraction, as well as increased STEMI prevalence [10]. Likewise, Lian et al.’s cross-sectional analysis in a Chinese ACS population showed that short sleep duration and poor sleep habits were linked to greater CAD severity via Gensini scores and higher myocardial infarct risk, with STEMI patients exhibiting more disrupted sleep patterns [16]. In contrast, mild sleep apnea has been proposed as a protective mechanism in myocardial infarcts through ischemic preconditioning [18]. Our data, focusing on post-ACS sleep quality in the elderly, contradict this potential link by showing that poor sleep is tied to worse biomarkers and STEMI dominance. This conflict might be explained by the variations in age from study to study [5]. We believe the present focus on geriatric-specific outcomes is a unique factor that identifies sleep quality as a modifiable factor that could influence ACS subtype severity and biomarker profiles. Poor sleep-related alterations in inflammatory functioning have indeed been described to be associated with ischemia–reperfusion injury through different mechanisms [27,28].

Sleep fragmentation is a hallmark of aging compounded by neurodegenerative changes in the suprachiasmatic nucleus [29,30]. Prolonged latency and inefficiency often signal underlying hyperarousal states, which can cause vascular inflammation and impair endothelial repair processes critical for CAD stabilization [9,14]. In our cohort, intermediate/high SYNTAX scores were associated with higher PSQI, shorter sleep duration (particularly 5–6 h), and lower efficiency. Lao et al., in their large prospective cohort of over 60,000 adults, demonstrated that short sleep duration (<6 h) and poor quality increased coronary risks, with a combined Sleep Score (integrating duration and quality) showing a 31% higher hazard for poorer sleep [4]. Similarly, Hoevenaar-Blom et al. reported that short sleep (≤6 h) was associated with 15% higher cardiovascular disease incidence [28], which is supported by tissue-level evidence [31]. Long sleep (>8 h), on the other hand, does not appear to constitute benefits [4], with other studies showing that poor sleep quality despite longer sleep duration (≥10 h) increases cardiovascular mortality [27]. Short duration and low efficiency may drive CAD progression via disrupted circadian regulation of lipid metabolism and heightened cortisol, which may have a dual impact due to elevated oxidative stress and reduced compensatory mechanisms [7,14].

Sleep disturbances may also be associated with various other factors, including metabolic and inflammatory markers [32]. In our multivariable logistic regression analysis, hypertension emerged as an independent predictor of high PSQI scores, alongside reduced ejection fraction. These associations suggest that systemic hypertension and ventricular dysfunction may also be linked to poor sleep (bidirectionally) in geriatric ACS. Matsuda et al. linked poor sleep quality in CVD patients to higher anxiety and depression scores, which often coexist with hypertension [27]. Askarinezhad et al. also noted that anxiety symptoms correlated with poor sleep in coronary angiography patients, potentially with mediating factors like female sex and lack of health insurance [31]. In a population-based cohort, Suzuki et al. demonstrated that poor sleep quality was associated with cardiovascular mortality and hypertension [28]. Our findings extend available knowledge by identifying hypertension and ejection fraction as independent factors associated with sleep metrics. We believe it would be beneficial to integrate sleep evaluations for elderly patients with ACS, as addressing sleep quality could enhance long-term prognosis.

This study has several notable limitations that warrant cautious interpretation of the findings. Primarily, its retrospective design causes reliance on existing medical records, which may lead to incomplete or inconsistent data on covariates like pre-ACS sleep habits or unmeasured confounders such as diet and physical activity which can impact sleep and CAD. Additionally, we did not systematically assess important potential confounders including depression and anxiety disorders, which are highly prevalent in elderly populations and strongly associated with both poor sleep quality and cardiovascular disease. Detailed alcohol consumption patterns, which can affect both sleep architecture and cardiovascular risk, were also not comprehensively evaluated. Furthermore, the study group was almost 92% male, and although male sex is a risk factor, this proportion is an overrepresentation relative to population-based data. The cross-sectional assessment of sleep quality at 6 months post-ACS precludes causal inference; we cannot determine whether poor sleep quality preceded or resulted from severe CAD, as the bidirectional nature of this relationship is well-established. PSQI assessment was performed at 6 months, which may not capture the characteristics of sleep linked to the event. Moreover, the PSQI is subjective and susceptible to recall bias or cognitive influences in geriatric patients. Objective measures like polysomnography (PSG) could have provided more precise data [10]. While the PSQI is well-validated in cardiovascular populations, objective sleep parameters such as sleep architecture, apnea-hypopnea index, and nocturnal oxygen desaturation would have provided more comprehensive characterization of sleep disturbances. Although patients with diagnosed obstructive sleep apnea (OSA) were excluded, systematic screening for undiagnosed OSA using polysomnography or home sleep apnea testing was not performed. Given the high prevalence of undiagnosed OSA in elderly populations and its strong association with CAD, occult sleep-disordered breathing may have confounded our findings. Furthermore, inflammatory biomarkers beyond C-reactive protein, such as interleukin-6 or tumor necrosis factor-alpha, were not measured, limiting our ability to explore mechanistic pathways linking sleep disturbance to atherosclerotic burden.

The single-center setting limits generalizability, as our tertiary hospital cohort may overrepresent severe cases, and regional factors could have skewed associations. The one-year follow-up may be insufficient to identify long-term CAD progression linked to persistent poor sleep. Additionally, the study population consisted exclusively of ACS survivors who reached 6-month follow-up, potentially introducing survival bias and excluding patients with the most severe disease who died before assessment. Finally, it is possible that cardiac events and poor sleep quality were bidirectionally associated, as suggested by prior research [2]. Despite these limitations, our findings provide valuable preliminary evidence for an association between sleep quality and CAD severity in geriatric ACS patients, highlighting the need for larger prospective studies with comprehensive sleep and psychological assessments.

## 5. Conclusions

This study demonstrates that poor sleep quality at six months post-event is independently associated with greater CAD severity, as evidenced by higher SYNTAX scores, alongside links to hypertension, reduced ejection fraction, elevated biomarkers, and STEMI predominance. Specific sleep deficits in duration and efficiency further correlate with complex coronary lesions, highlighting sleep’s role in post-ACS vascular burden. These findings underscore the need for routine sleep assessments in elderly ACS management, potentially informing targeted interventions to mitigate CAD progression and improve outcomes in this high-risk group. Clinically, integrating sleep quality screening into post-ACS care pathways may help identify higher-risk geriatric patients. Targeted interventions to improve sleep—including cognitive-behavioral therapy for insomnia, sleep hygiene education, and optimization of medication timing—may represent cost-effective adjunctive strategies to reduce cardiovascular burden. Future randomized trials should determine whether sleep-focused interventions improve cardiovascular outcomes in elderly ACS survivors.

## Figures and Tables

**Table 1:** Summary of patients’ and sleep characteristics and laboratory measurements with regard to PSQI global score.

		PSQI Global Score		
	Total (n = 308)	≤5 (n = 176)	>5 (n = 132)	*p*
Age, years	67 (65–73)	67 (65–73)	69 (65–75)	**0.049 ^‡^**
Sex				
Female	25 (8.12%)	16 (9.09%)	9 (6.82%)	0.609 ^§^
Male	283 (91.88%)	160 (90.91%)	123 (93.18%)	
Body mass index, kg/m^2^	27.83 ± 4.09	27.64 ± 3.96	28.08 ± 4.27	0.355 ^†^
Hypertension	135 (43.83%)	68 (38.64%)	67 (50.76%)	**0.034 ^§^**
Diabetes mellitus	85 (27.60%)	41 (23.30%)	44 (33.33%)	0.051 ^§^
Stroke	5 (1.62%)	3 (1.70%)	2 (1.52%)	1.000 ^#^
Atrial fibrillation	7 (2.27%)	3 (1.70%)	4 (3.03%)	0.467 ^#^
Thyroid disease	17 (5.52%)	8 (4.55%)	9 (6.82%)	0.540 ^§^
COPD	28 (9.09%)	15 (8.52%)	13 (9.85%)	0.841 ^§^
Sleep apnea	31 (10.06%)	15 (8.52%)	16 (12.12%)	0.397 ^§^
LVEF, %	48.00 ± 8.50	49.51 ± 8.44	46.00 ± 8.20	**<0.001 ^†^**
Diuretics use	47 (15.26%)	25 (14.20%)	22 (16.67%)	0.664 ^§^
ACE inhibitors/ARB use	70 (22.73%)	37 (21.02%)	33 (25.00%)	0.410 ^§^
Beta blockers use	40 (12.99%)	22 (12.50%)	18 (13.64%)	0.903 ^§^
Calcium channel blockers use	18 (5.84%)	12 (6.82%)	6 (4.55%)	0.551 ^§^
Smoking	101 (32.79%)	57 (32.39%)	44 (33.33%)	0.861 ^§^
Living alone	66 (21.43%)	35 (19.89%)	31 (23.48%)	0.446 ^§^
Acute coronary syndrome				
NSTEMI	104 (33.77%)	63 (35.80%)	41 (31.06%)	**0.016 ^§^**
STEMI	154 (50.00%)	77 (43.75%)	77 (58.33%) *	
USAP	50 (16.23%)	36 (20.45%)	14 (10.61%) *	
Revascularization				
Medical	28 (9.09%)	18 (10.23%)	10 (7.58%)	0.712 ^§^
PCI	249 (80.84%)	140 (79.55%)	109 (82.58%)	
CABG	31 (10.06%)	18 (10.23%)	13 (9.85%)	
SYNTAX score	17.88 ± 8.20	15.94 ± 7.53	20.46 ± 8.36	**<0.001 ^†^**
Low (≤22)	210 (68.18%)	137 (77.84%)	73 (55.30%) *	**<0.001 ^§^**
Intermediate (23–32)	85 (27.60%)	36 (20.45%)	49 (37.12%) *	
High (≥33)	13 (4.22%)	3 (1.70%)	10 (7.58%) *	
Hemoglobin, g/dL	13.89 ± 1.87	13.91 ± 1.81	13.85 ± 1.95	0.777 ^†^
Platelet, ×10^3^	234.5 (196.5–285.5)	232 (192–279.5)	236 (197.5–290)	0.500 ^‡^
WBC, ×10^3^	10.57 ± 3.23	10.38 ± 3.05	10.82 ± 3.44	0.236 ^†^
Creatinine, mg/dL	0.90 (0.78–1.05)	0.87 (0.78–1.02)	0.94 (0.78–1.10)	0.085 ^‡^
eGFR, mL/min/1.73 m^2^	83.97 ± 22.81	86.50 ± 21.99	80.61 ± 23.54	**0.025 ^†^**
Uric acid, mg/dL	5.7 (4.8–6.5)	5.7 (4.5–6.45)	5.7 (4.8–6.55)	0.732 ^‡^
CRP, mg/L	4.65 (3.1–9.5)	4.05 (3.0–8.6)	5.1 (3.1–10.2)	**0.029 ^‡^**
Total cholesterol, mg/dL	178.74 ± 39.58	179.49 ± 38.51	177.74 ± 41.08	0.701 ^†^
HDL, mg/dL	40.51 ± 10.44	40.20 ± 9.07	40.92 ± 12.05	0.570 ^†^
LDL, mg/dL	112.50 ± 35.52	113.36 ± 34.90	111.36 ± 36.44	0.627 ^†^
Triglyceride, mg/dL	115.5 (78–181.5)	114 (76–186)	117.5 (78–176)	0.917 ^‡^
Fasting blood glucose, mg/dL	107 (94–133)	107 (94–131)	107 (94–137)	0.728 ^‡^
Peak troponin, ng/L	3.960 (1.300–16.005)	3.760 (1.245–14.000)	4.390 (1.545–19.795)	0.490 ^‡^
NT-proBNP, pg/mL	665.5 (436.5–1089)	595 (416–1033)	748.5 (453.5–1316)	**0.034 ^‡^**
Sleep latency				
<15 min	109 (35.39%)	93 (52.84%)	16 (12.12%) *	**<0.001 ^§^**
16–30 min	126 (40.91%)	71 (40.34%)	55 (41.67%)	
31–60 min	57 (18.51%)	11 (6.25%)	46 (34.85%) *	
>60 min	16 (5.19%)	1 (0.57%)	15 (11.36%) *	
Sleep duration				
>7 h	138 (44.81%)	112 (63.64%)	26 (19.70%) *	**<0.001 ^§^**
6–7 h	90 (29.22%)	54 (30.68%)	36 (27.27%)	
5–6 h	64 (20.78%)	10 (5.68%)	54 (40.91%) *	
<5 h	16 (5.19%)	0 (0.00%)	16 (12.12%) *	
Sleep efficiency				
≥85%	179 (58.12%)	145 (82.39%)	34 (25.76%) *	**<0.001 ^§^**
75–84%	79 (25.65%)	28 (15.91%)	51 (38.64%) *	
65–74%	37 (12.01%)	3 (1.70%)	34 (25.76%) *	
<65%	13 (4.22%)	0 (0.00%)	13 (9.85%) *	

Descriptive statistics are presented using mean ± standard deviation for normally distributed continuous variables, median (25th percentile–75th percentile) for non-normally distributed continuous variables and frequency (percentage) for categorical variables. ^†^ Student’s t test, ^‡^ Mann–Whitney U test, ^§^ Chi-square test, ^#^ Fisher’s exact test, * Statistically significant category for the variables with three or more categories. Statistically significant *p* values are shown in bold. Abbreviations: ACE: Angiotensin-Converting Enzyme, ARB: Angiotensin Receptor Blocker, BMI: Body Mass Index, CABG: Coronary Artery Bypass Grafting, COPD: Chronic Obstructive Pulmonary Disease, CRP: C-Reactive Protein, eGFR: Estimated Glomerular Filtration Rate, HDL: High-Density Lipoprotein, LDL: Low-Density Lipoprotein, LVEF: Left Ventricular Ejection Fraction, NSTEMI: Non–ST-Elevation Myocardial Infarction, NT-proBNP: N-terminal pro–B-type Natriuretic Peptide, PCI: Percutaneous Coronary Intervention, PSQI: Pittsburgh Sleep Quality Index, STEMI: ST-Elevation Myocardial Infarction, SYNTAX: Synergy Between PCI With Taxus and Cardiac Surgery, USAP: Unstable Angina Pectoris, WBC: White Blood Cell.

**Table 2:** Independent associations between factors and high (>5) PSQI score, multivariable logistic regression analysis.

	β Coefficient	Standard Error	*p*	Exp (β)	95% CI for Exp (β)		VIF
Age	0.019	0.023	0.392	1.020	0.975	1.066	1.363
Hypertension, Yes ^(1)^	0.679	0.267	**0.011**	1.971	1.168	3.328	1.122
Ejection fraction	−0.052	0.024	**0.030**	0.949	0.905	0.995	2.636
Acute coronary syndrome ^(2)^							
NSTEMI	0.432	0.400	0.281	1.540	0.703	3.374	2.134
STEMI	0.766	0.381	**0.045**	2.151	1.019	4.542	2.210
SYNTAX score ^(3)^							
Intermediate (23–32)	0.853	0.282	**0.003**	2.347	1.350	4.082	1.089
High (≥33)	1.846	0.711	**0.009**	6.336	1.573	25.514	1.045
Glomerular filtration rate	−0.003	0.006	0.619	0.997	0.984	1.009	1.384
CRP	−0.001	0.006	0.899	0.999	0.988	1.010	1.103
NT-proBNP	0.000	0.000	0.447	1.000	0.999	1.000	2.472
Constant	0.155	2.228	0.944	1.168			

Nagelkerke R^2^ = 0.175, CI: Confidence interval, VIF: Variance inflation factor. Statistically significant *p* values are shown in bold. ^(1)^ Reference category is “No hypertension”, ^(2)^ Reference category is “USAP”, ^(3)^ Reference category is “Low (≤22)”. Abbreviations; CI: Confidence Interval, CRP: C-Reactive Protein, eGFR: Estimated Glomerular Filtration Rate, NSTEMI: Non–ST-Elevation Myocardial Infarction, NT-proBNP: N-terminal pro–B-type Natriuretic Peptide, PSQI: Pittsburgh Sleep Quality Index, STEMI: ST-Elevation Myocardial Infarction, SYNTAX: Synergy Between PCI With Taxus and Cardiac Surgery, USAP: Unstable Angina Pectoris.

**Table 3:** Summary of sleep characteristics with regard to SYNTAX score.

	SYNTAX Score		
	Low, ≤22 (n = 210)	Intermediate & High, >22 (n = 98)	*p*
PSQI global score	4 (3–6)	6 (4–9)	**<0.001 ^‡^**
≤5	137 (65.24%)	39 (39.80%)	**<0.001 ^§^**
>5	73 (34.76%)	59 (60.20%)	
Sleep latency			
<15 min	83 (39.52%)	26 (26.53%)	0.129 ^§^
16–30 min	82 (39.05%)	44 (44.90%)	
31–60 min	34 (16.19%)	23 (23.47%)	
>60 min	11 (5.24%)	5 (5.10%)	
Sleep duration			
>7 h	106 (50.48%)	32 (32.65%) *	**0.004 ^§^**
6–7 h	59 (28.10%)	31 (31.63%)	
5–6 h	33 (15.71%)	31 (31.63%) *	
<5 h	12 (5.71%)	4 (4.08%)	
Sleep efficiency			
≥85%	139 (66.19%)	40 (40.82%) *	**<0.001 ^§^**
75–84%	45 (21.43%)	34 (34.69%) *	
65–74%	18 (8.57%)	19 (19.39%) *	
<65%	8 (3.81%)	5 (5.10%)	

Descriptive statistics are presented using median (25th percentile–75th percentile) for non-normally distributed continuous variables and frequency (percentage) for categorical variables. ^‡^ Mann–Whitney U test, ^§^ Chi-square test, * Statistically significant category for the variables with three or more categories. Statistically significant *p* values are shown in bold. Abbreviations; PSQI: Pittsburgh Sleep Quality Index, SYNTAX: Synergy Between PCI With Taxus and Cardiac Surgery.

## Data Availability

Data supporting the findings of this study are available from the corresponding author upon reasonable request.

## Abbreviations

CAD	coronary artery disease
ACS	acute coronary syndrome
PSQI	Pittsburgh Sleep Quality Index
STEMI	ST-elevation myocardial infarction
NSTEMI	non-ST-elevation myocardial infarction
USAP	unstable angina pectoris
OSA	obstructive sleep apnea
MACE	major adverse cardiovascular events
